# The Two-Component Response Regulator Ssk1 and the Mitogen-Activated Protein Kinase Hog1 Control Antifungal Drug Resistance and Cell Wall Architecture of Candida auris

**DOI:** 10.1128/mSphere.00973-20

**Published:** 2020-10-14

**Authors:** Raju Shivarathri, Sabrina Jenull, Anton Stoiber, Manju Chauhan, Rounik Mazumdar, Ashutosh Singh, Filomena Nogueira, Karl Kuchler, Anuradha Chowdhary, Neeraj Chauhan

**Affiliations:** a Public Health Research Institute, Rutgers, The State University of New Jersey, Newark, New Jersey, USA; b Department of Microbiology, Biochemistry and Molecular Genetics, New Jersey Medical School, Rutgers, The State University of New Jersey, Newark, New Jersey, USA; c Medical University of Vienna, Department of Medical Biochemistry, Max Perutz Labs Vienna, Vienna, Austria; d Department of Medical Mycology, Vallabhbhai Patel Chest Institute, University of Delhi, Delhi, India; e CCRI—St. Anna Children's Cancer Research Institute, Vienna, Austria; f Labdia—Labordiagnostik GmbH, Vienna, Austria; University of Georgia

**Keywords:** *Candida auris*, SSK1, HOG1, multidrug resistance, cell wall, MAPK signaling, caspofungin, amphotericin B, stress response

## Abstract

Candida auris is an emerging multidrug-resistant human fungal pathogen refractory to treatment by several classes of antifungal drugs. Unlike other *Candida* species, C. auris can adhere to human skin for prolonged periods of time, allowing for efficient skin-to-skin transmission in the hospital environments. However, molecular mechanisms underlying pronounced multidrug resistance and adhesion traits are poorly understood. Two-component signal transduction and mitogen-activated protein (MAP) kinase signaling are important regulators of adherence, antifungal drug resistance, and virulence. Here, we report that genetic removal of *SSK1* encoding a response regulator and the mitogen-associated protein kinase *HOG1* restores the susceptibility to both amphotericin B (AMB) and caspofungin (CAS) in C. auris clinical strains. The loss of *SSK1* and *HOG1* alters membrane lipid permeability, cell wall mannan content, and hyperresistance to cell wall-perturbing agents. Interestingly, our data reveal variable functions of *SSK1* and *HOG1* in different C. auris clinical isolates, suggesting a pronounced genetic plasticity affecting cell wall function, stress adaptation, and multidrug resistance. Taken together, our data suggest that targeting two-component signal transduction systems could be suitable for restoring C. auris susceptibility to antifungal drugs.

**IMPORTANCE** Candida auris is an emerging multidrug-resistant (MDR) fungal pathogen that presents a serious global threat to human health. The Centers for Disease Control and Prevention (CDC) have classified C. auris as an urgent threat to public health for the next decade due to its major clinical and economic impact and the lack of effective antifungal drugs and because of future projections concerning new C. auris infections. Importantly, the Global Antimicrobial Resistance Surveillance System (GLASS) has highlighted the need for more robust and efficacious global surveillance schemes enabling the identification and monitoring of antifungal resistance in *Candida* infections. Despite the clinical relevance of C. auris infections, our overall understanding of its pathophysiology and virulence, its response to human immune surveillance, and the molecular basis of multiple antifungal resistance remains in its infancy. Here, we show a marked phenotypic plasticity of C. auris clinical isolates. Further, we demonstrate critical roles of stress response mechanisms in regulating multidrug resistance and show that cell wall architecture and composition are key elements that determine antifungal drug susceptibilities. Our data promise new therapeutic options to treat drug-refractory C. auris infections.

## Abstract (precis)

Candida auris is an emerging multidrug-resistant (MDR) fungal pathogen that presents a serious global threat to human health. The Centers for Disease Control and Prevention (CDC) have classified C. auris as an urgent threat to public health for the next decade due to its major clinical and economic impact and the lack of effective antifungal drugs and because of future projections concerning new C. auris infections. Importantly, the Global Antimicrobial Resistance Surveillance System (GLASS) has highlighted the need for more robust and efficacious global surveillance schemes enabling the identification and monitoring of antifungal resistance in *Candida* infections. Despite the clinical relevance of C. auris infections, our overall understanding of its pathophysiology and virulence, its response to human immune surveillance, and the molecular basis of multiple antifungal resistance remains in its infancy. Here, we show a marked phenotypic plasticity of C. auris clinical isolates. Further, we demonstrate critical roles of stress response mechanisms in regulating multidrug resistance and show that cell wall architecture and composition are key elements that determine antifungal drug susceptibilities. Our data promise new therapeutic options to treat drug-refractory C. auris infections.

## INTRODUCTION

Invasive fungal infections constitute a staggering impact on global human health, claiming approximately 1.5 million lives a year worldwide (1). This number is estimated to continue to rise due to increasing numbers of immunosuppressed patient cohorts (2). *Candida* species, unlike most other major fungal pathogens, are normal commensal colonizers of human barrier microbiota, present on mucosal surfaces and in the gastrointestinal tract in healthy individuals (3). *Candida* spp. are a pivotal part of mycobiomes that are formed by hundreds of additional fungal species in a tissue-specific manner (4). Importantly, immune suppression can promote a *Candida* species to switch from being a benign commensal to being an invasive pathogen, leading to life-threatening systemic disease (3). Overall, *Candida* spp. cause about 400,000 bloodstream infections annually worldwide, with an associated mortality rate of 46 to 75%, which essentially has remained unchanged for decades (1).

Candida auris is an emerging nosocomial pathogen that causes outbreaks in intensive care units (ICUs) worldwide as well as infections in elderly patients, especially those who are in long-term nursing care facilities in the United States (5). The currently known C. auris clades I to V (6, 7) may have a common ancestor that likely occurred within the last 360 years (8). Strikingly, the rapid appearance of multidrug- or panresistant (all three classes of antifungals, i.e., amphotericin B [AMB], azoles, and echinocandins) C. auris clinical isolates (9, 10) has been sparking serious medical concerns, since treatment options for C. auris infections have become very limited. Thus, the limited number of antifungal drug classes, together with the increasing prevalence of bloodstream fungal infections and the emerging antifungal multidrug resistance (MDR) in C. auris, underscores the critical need for new and more effective antifungals.

The haploid genome of C. auris is estimated at around 12 Mb, distributed over seven chromosomes (9, 11). Genome sequencing shows that the C. auris genome harbors genes and pathways conserved in most fungal pathogens, including the two-component signal transduction system and mitogen-activated protein (MAP) kinase (MAPK) signaling pathway(s). Of note, these genes are implicated in both drug resistance and virulence in other *Candida* spp. (9). Two-component signaling pathways function by transferring phosphoryl groups among their components using a phosphorelay engaging aspartate or histidine residues accepting phosphoryl groups. The term “two-component” signaling was first coined for bacterial systems, where these phosphorelays engage only two proteins (12). Fungal two-component systems (TCS) involve three proteins: a histidine kinase, a phosphotransferase, and a response regulator operating in a linear manner (13). The activated response regulator frequently activates a downstream MAP kinase signaling cascade, which, in turn, controls dedicated transcription factors associated with morphogenesis, adhesion, stress response, drug resistance, and virulence (14–19). Although fungal TCS are often not essential for viability, multiple studies demonstrate their critical role in regulating virulence of many fungal pathogens (20–27). Hence, targeting TCS function holds promises for the development of new antifungal drugs with broad pathogen spectra. Importantly, TCS are found only in bacteria, plants, and fungi but not in humans (28), suggesting that the pharmacological targeting could avoid significant off-target toxicity effects for the host. Based on their function in pathogens, we reasoned that the C. auris TCS response regulator Ssk1 and the downstream MAP kinase Hog1 play essential roles in the regulation of antifungal MDR and cell wall function.

Here, we show that Ssk1 and Hog1 control resistance to both caspofungin (CAS) and amphotericin B (AMB), as genetic ablation of Ssk1 or Hog1 fully abrogates AMB and CAS resistance. Moreover, *hog1*Δ cells display reduced thermotolerance with an inability to grow at 42°C. Furthermore, the phenotypic analysis of both *ssk1*Δ and *hog1*Δ mutants in several clinical strain backgrounds from the African and Asian clades suggests important roles in controlling cell wall integrity and surface architecture, as well as the ability to adapt to osmotic, oxidative, and antifungal stress. Finally, Ssk1 and Hog1 functions appear variable in distinct C. auris clinical strain backgrounds, demonstrating a marked phenotypic plasticity of C. auris, which is most likely due to adaptive cell wall alterations, which also drive antifungal MDR phenotypes. Indeed, our data suggest the adaptive potential of C. auris engages TCS function and regulates complex signaling cross talk of MAPK pathways governing cell integrity, cell wall function, osmostress, and morphogenesis. Our work suggests that the TCS in C. auris may pave the way for efficient personalized antifungal strategies aimed at resensitizing drug-resistant C. auris infections in therapeutic settings.

## RESULTS

### *SSK1* and *HOG1* encode a putative two-component response regulator and a MAP kinase.

**FIG 1:**
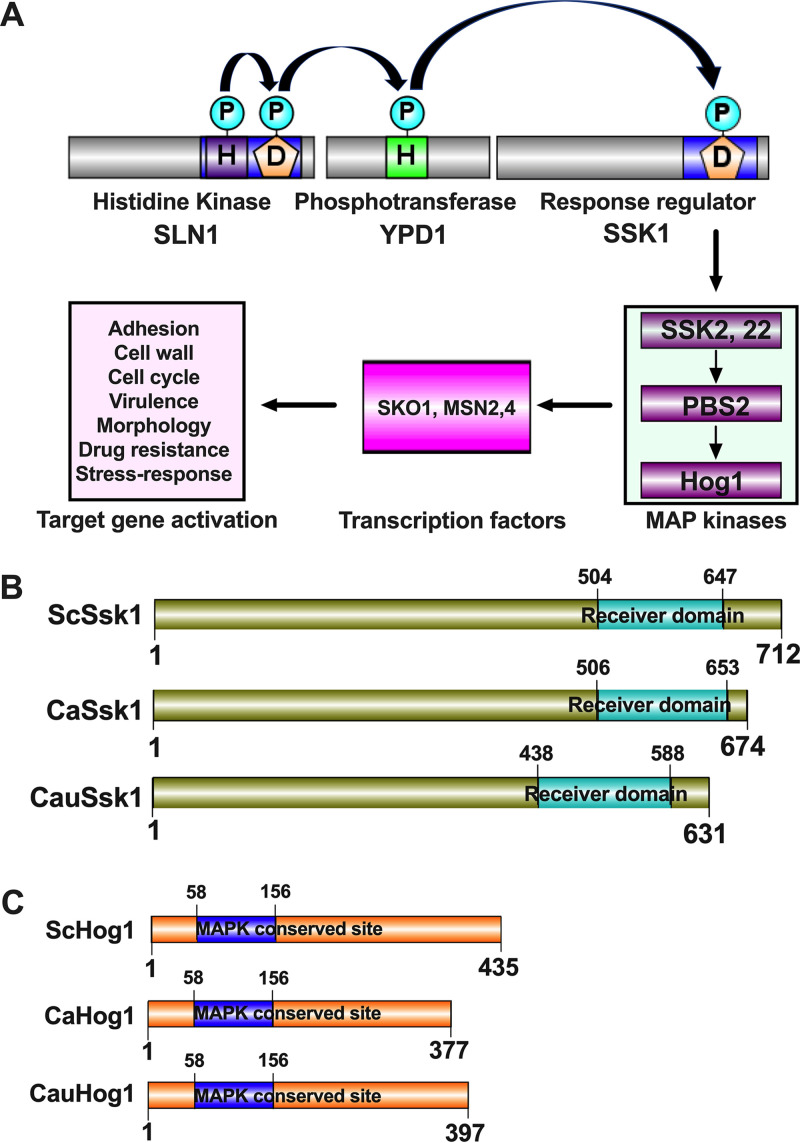
Two-component phosphorelay signaling system. (A) Fungal two-component signaling includes a multistep His-to-Asp phosphorelay in response to environmental cues. Sensing external stimuli leads to autophosphorylation of the sensor histidine kinase at the receiver domain (phosphoryl transfer between a histidine residue and an aspartate residue), followed by sequential phosphorelays between an aspartate residue (D) in the sensor protein, a histidine residue (H) in the intermediate phosphotransferase, and an aspartate residue (D) in the receiver domain of the response regulator (RR). Active RR activates downstream MAPK signaling, which modulates transcription factors and target genes eliciting cellular responses. (B and C) Schematic illustration of domains of Ssk1 and Hog1 proteins predicted to possess a C-terminal receiver domain and a conserved N-terminal MAPK domain, respectively. Domain prediction used the bioinformatics tool InterPro (https://www.ebi.ac.uk/interpro/). Ssk1 and Hog1 domain arrangements were generated by using online software IBS, an illustrator for the presentation and visualization of biological sequences.

The Candida auris *SSK1* (B9J08_005450) and *HOG1* (B9J08_004369) genes contain open reading frames of 1,896 and 1,194 nucleotides, putatively encoding the 69.6-kDa Ssk1 and 45.09-kDa Hog1 kinase, respectively. Ssk1 contains a prototypical receiver domain, while Hog1 contains a conserved serine-threonine kinase motif found in other fungal Hog1 kinases (Fig. 1). Alignment of C. auris Ssk1 with Candida albicans and Saccharomyces cerevisiae Ssk1 revealed 34% and 24% identity (data not shown), respectively. Of note, C. auris Hog1 had an even higher conservation to C. albicans and S. cerevisiae Hog1 orthologues, sharing 82% and 70% similarities, respectively (data not shown). Of note, as for Ssk1, similarities were confined to the C-terminal receiver domain, in which D488 is a putative phosphorylation site (data not shown).

**FIG 2:**
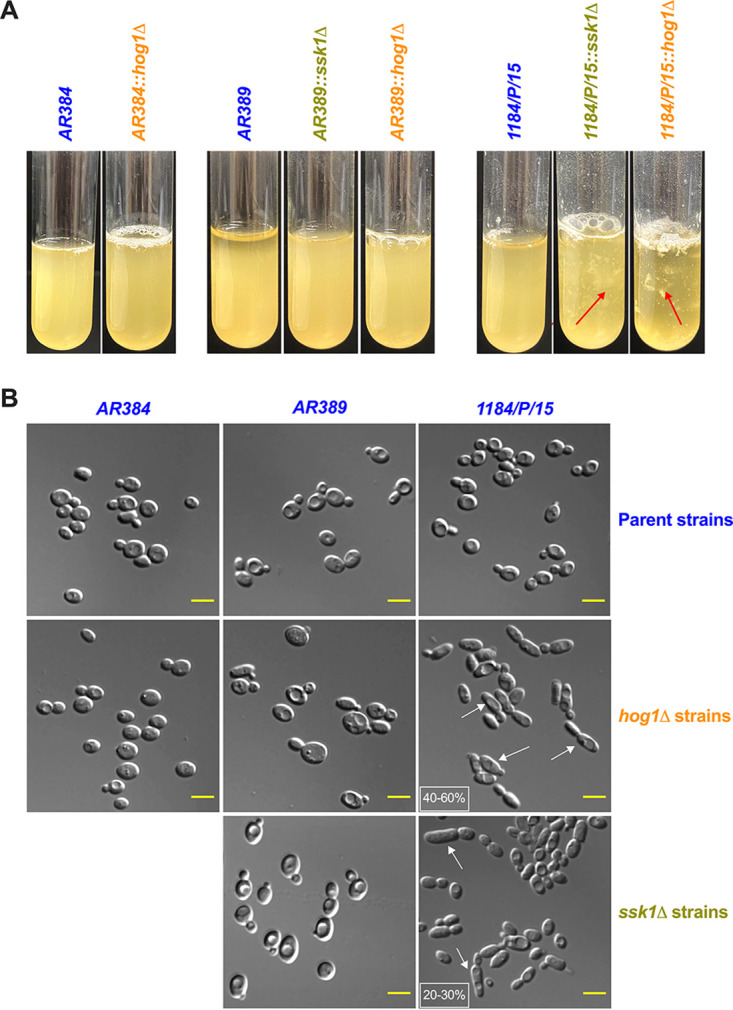
Phenotypic profiling of Candida auris clinical isolates. (A) Flocculation of *ssk1*Δ and *hog1*Δ mutants in different parental strain backgrounds was tested. All the samples were vortexed and photographed after 5 min. Red arrows indicate the floccules in the suspension. (B) Representative microscopy differential interference contrast (DIC) images showing the elongated pseudohyphal morphology of indicated strains. Logarithmically growing cells in YPD were washed, and images were taken with a Zeiss Axiovert 200 microscope at ×63 magnification. White arrows indicate the elongated cells, and the percentage of these cells was depicted in the small white box (scale bar = 5 μm).

**FIG 3:**
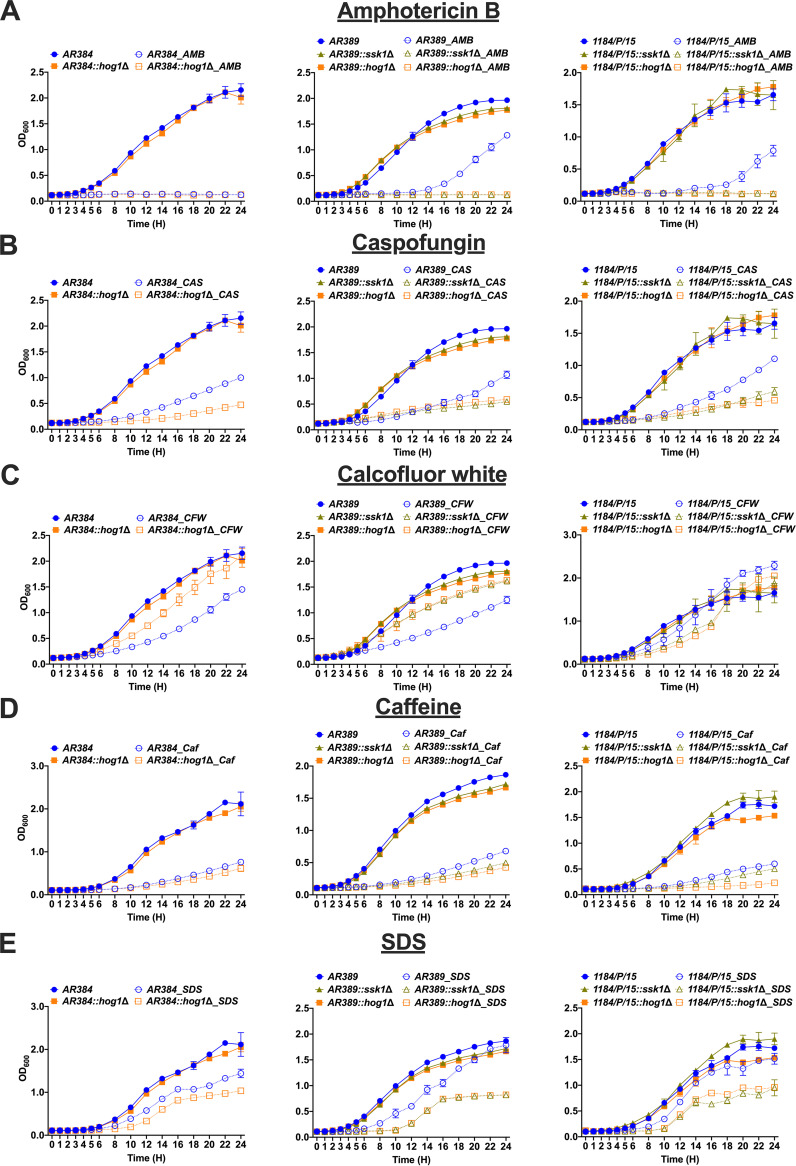
Lack of *SSK1* and *HOG1* leads to altered susceptibility to antifungal drugs and cell wall stress agents. Liquid growth of indicated strains at 30°C in YPD medium containing various antifungal drugs and cell wall stress agents as indicated. (A to E) Amphotericin B (AMB, 500 ng/ml), caspofungin (CAS, 100 ng/ml), calcofluor white (CFW, 50 μg/ml), caffeine (Caf, 50 mM), and SDS (0.05%), respectively. Data represent the mean (±SD) from three independent experiments. Parent strains and *ssk1*Δ and *hog1*Δ strains are indicated in blue, light green, and orange lines, respectively. Cultures grown in YPD and antifungal drug or cell wall stress agent are represented with solid lines and dotted lines, respectively.

To assess a possible role of the TCS components Ssk1 and Hog1 in antifungal resistance, we created deletion strains lacking *SSK1* and *HOG1* in the AR389 and 1184/P/15 strains. In addition, we also deleted *HOG1* in AR384, but despite repeated efforts, we were unable to obtain a mutant strain lacking *SSK1* in AR384. Strain AR384 is from the African clade, while the strains AR389 and 1184/P/15 belong to the South Asian clade (see Table S1 in the supplemental material). We slightly modified the fusion PCR method (29) to generate deletion constructs containing ∼500 bp of homologous upstream (5′ untranslated region [UTR]) and downstream (3′ UTR) homologous flanking regions, fused to the dominant marker *NAT1* and flanked by constant overlapping sequences U1 and D1, respectively (Fig. S1). The resulting knockout strains and the isogenic parental strains were subjected to extensive phenotypic profiling to test for morphogenesis, antifungal susceptibility, and sensitivity to stress and cell wall-perturbing agents (Fig. 2 and 3 and Fig. S2).

10.1128/mSphere.00973-20.1 FIG S1 Schematic illustration of fusion-PCR-based gene deletion strategy in C. auris. Generation of *ssk1Δ* and *hog1Δ* gene deletion constructs by fusion PCR using the dominant selectable marker *NAT1*. Two unique overlapping sequences (U1 and D1) were integrated in oligonucleotides to amplify the *NAT1* marker. The resulting fusion products after 4 rounds of PCR (upper *in vitro* panel) were gel purified and transformed into drug-resistant C. auris clinical isolates (lower *in vivo* panel). Genomic DNA prepared from transformants was verified by colony PCR for correct integration at the 5′ (5C) and 3′ (3C) junctions and checked for loss of the target gene (Log_F/Log_R). Download FIG S1, TIF file, 1.0 MB. Copyright © 2020 Shivarathri et al. 2020 Shivarathri et al. This content is distributed under the terms of the Creative Commons Attribution 4.0 International license.

10.1128/mSphere.00973-20.2 FIG S2 Deletion of *SSK1* and *HOG1* leads to altered susceptibility to antifungal drugs, cell wall stress and oxidative stress. (A and B) Equal volumes (3 μl) of 10-fold serial dilutions of logarithmically growing C. auris strains were spotted onto YPD plates containing different stress agents such as antifungal stress (caspofungin [CAS, 200 ng/ml], amphotericin B [AMB, 0.5 and 2 μg/ml], and fluconazole [FLC, 16 and 128 μg/ml]), thermal stress (42°C), cell wall stress (caffeine [50 mM], SDS [0.05%], and calcofluor white [CFW, 50 μg/ml]), osmostress (sodium chloride [NaCl, 1 M]), and oxidative stress (hydrogen peroxide [H_2_O_2_, 10 mM]). Colony growth was scored after 2 days and compared to the YPD control plate. Download FIG S2, TIF file, 1.7 MB. Copyright © 2020 Shivarathri et al. 2020 Shivarathri et al. This content is distributed under the terms of the Creative Commons Attribution 4.0 International license.

10.1128/mSphere.00973-20.6 TABLE S1 Candida auris and Candida albicans strains used in this study. Download Table S1, DOCX file, 0.02 MB. Copyright © 2020 Shivarathri et al. 2020 Shivarathri et al. This content is distributed under the terms of the Creative Commons Attribution 4.0 International license.

### Lack of *SSK1* and *HOG1* results in increased flocculation and formation of elongated cell morphologies.

To determine the effect of disruption of *SSK1* and *HOG1* in C. auris, the *ssk1Δ* and *hog1Δ* null mutants along with their parental strains were grown in yeast extract-peptone-dextrose (YPD) broth at 30°C. We noticed that both *ssk1Δ* and *hog1Δ* mutant strains flocculated extensively in the strain 1184/P/15 background (Fig. 2A). Interestingly, deletion of *SSK1* and *HOG1* in strains AR384 and AR389 did not result in any noticeable flocculation (Fig. 2A). These data suggest a variable function of Ssk1 and Hog1 in controlling cell surface function in different C. auris strains. Furthermore, microscopic inspection of *ssk1Δ* and *hog1Δ* mutants in the 1184/P/15 background revealed elongated cell shapes compared to the parental strain (Fig. 2B). The *ssk1Δ* and *hog1Δ* mutants in the AR384 and AR389 background did not show any obvious changes in cell morphology (Fig. 2B).

### Deletion of *SSK1* and *HOG1* alters susceptibilities of C. auris strains to CAS and AMB.

The majority of C. auris clinical isolates display pronounced antifungal MDR phenotypes (10). According to recent CDC estimates, 90% of all C. auris isolates are resistant to at least one antifungal, while 30% of isolates are resistant to at least two antifungals (https://www.cdc.gov/drugresistance/pdf/threats-report/2019-ar-threats-report-508.pdf). In addition, panresistant C. auris strains refractory to relevant antifungals have been emerging within all clades (10). AMB resistance is a cause for serious concern, since AMB resistance is rare in *Candida* spp. (30). AMB resistance essentially eliminates the last therapeutic option to treat fungal pathogens (31). Remarkably, the parental AR389 and 1184/P/15 strains displayed pronounced resistance to AMB, fluconazole (FLC), and CAS resistance phenotypes (Fig. 3 and Fig. S2). The AR384 isolate was AMB sensitive, but AR389 and 1184/P/15 showed elevated basal tolerance to AMB and CAS (Fig. 3 and Fig. S2). Of note, 1184/P/15 was unable to grow at 42°C and it was hypersensitive to peroxide stress (Fig. S2). However, deletion of both *SSK1* and *HOG1* in AR384, AR389, and 1184/P/15 strains restored sensitivity to both AMB and CAS (Fig. 3 and Fig. S2).

### C. auris *ssk1*Δ and *hog1*Δ mutants display distinct susceptibilities to cell wall-disrupting agents.

The fungal cell wall is the first point of contact between the host immune cells and the pathogen, and its integrity and architecture mediate immune recognition (32, 33). Therefore, we sought to determine how deleting *SSK1* and *HOG1* would impact cell wall function. Thus, we treated parental strains and single mutants with cell wall-disrupting agents, including SDS, caffeine, and calcofluor white (CFW) (Fig. 3 and Fig. S2). Further, we also assessed growth phenotypes in the absence and presence of antifungal drugs (Fig. 3 and Fig. S2). Surprisingly, the phenotypic analysis (Fig. 3 and Fig. S2) revealed significant differences in the ability of distinct strains to adapt to elevated temperature, oxidative stress, and susceptibility to cell wall damage. *ssk1*Δ and *hog1*Δ mutants showed increased resistance to CFW (Fig. 3 and Fig. S2), which is known to affect chitin distribution in fungal cell walls (34). These data suggest that *ssk1*Δ and *hog1*Δ mutants may hold cell wall alterations that affect function and/or surface architecture. Indeed, both deletion mutants were also sensitive to SDS, as well as to caffeine (Fig. 3 and Fig. S2), known inhibitors/activators of the cell wall integrity pathway in fungi (35, 36). The most profound effects were observed in 1184/P/15, where the deletion of *SSK1* and *HOG1* rendered C. auris hypersensitive to both cell wall stress and osmostress. The data confirm that Ssk1 and Hog1 exert strain-specific and distinct roles concerning cell wall functions and drug susceptibility in different clinical isolates. Moreover, the data show remarkable phenotypic diversities among clinical C. auris strains both within and between different clades.

### Genetic removal of *SSK1* and *HOG1* alters membrane lipid permeability.

**FIG 4:**
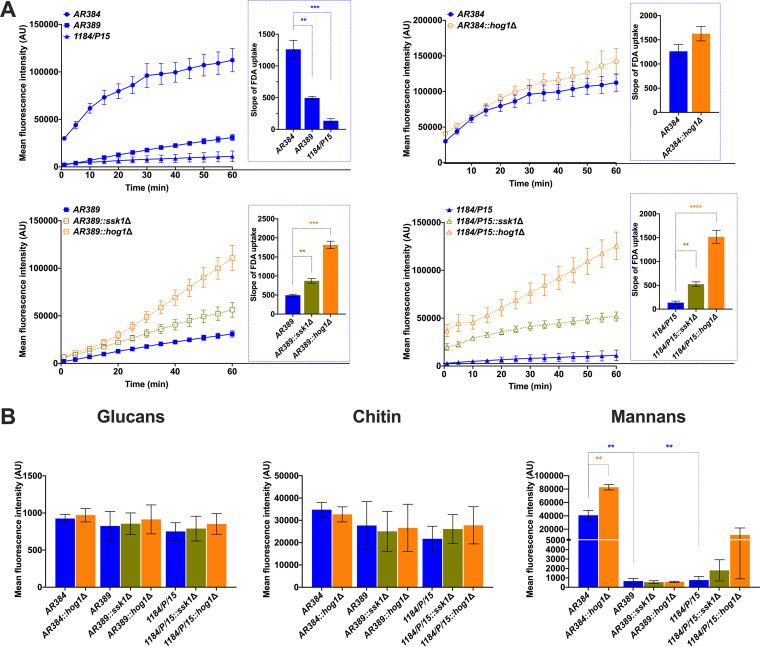
Genetic removal of *SSK1* and *HOG1* alters the membrane permeability. (A) The kinetics of fluorescence-based fluorescein diacetate (FDA) uptake was measured as described in Materials and Methods. FDA uptake was allowed with continuous shaking; OD reads were taken every 5 min for 30 reads or until saturation was reached. Data represent the mean fluorescence intensity from three independent experiments (±SEM; **, *P* < 0.01; ***, *P* < 0.0005). The slope was calculated using GraphPad Prism and shown in the graph (right side inset). (B) Flow cytometry-based quantification of cell wall components in Candida auris. Logarithmically growing *Candida* cultures were washed and triple stained to decorate cell wall components before quantification of β-d-glucan (FITC), chitin (BV421), and mannan (Texas Red) in suitable laser channels. Data represent the mean fluorescence intensity (±SEM; *, *P* < 0.05; **, *P* < 0.005) from three biological replicates.

To determine the potential cause of changing drug susceptibility of *ssk1*Δ and *hog1*Δ mutants, we reasoned that clinical strains have distinct membrane lipid features that affect non-protein-mediated drug permeability. Hence, we measured the kinetics of fluorescein diacetate (FDA) uptake by C. auris strains. The FDA is a lipophilic nonfluorescent precursor, which liberates the fluorescent dye after cleavage by an intracellular esterase (37). FDA uptake by the yeast cells is driven by passive diffusion, and uptake kinetics is determined by the lipid fluidity that determines membrane permeability. Indeed, the data showed higher membrane permeability of *ssk1*Δ and *hog1*Δ mutants than of the isogenic parental strains showing slower FDA uptake, especially the 1184/P/15 strain (Fig. 4A). Remarkably, FDA uptake into *ssk1*Δ and *hog1*Δ mutants was much faster as reflected by the higher slope of appearing fluorescence. These data correlated with the restored susceptibility to AMB and CAS. Further, the data suggest that membrane lipid permeability changes provide a major control element for diffusional uptake of antifungals. Indeed, TCS and MAPK signaling are implicated in regulating membrane permeability. Moreover, our data indicate that the C. auris Ssk1 TCS and the downstream Hog1 MAP kinase pathway cooperate in the regulation of sensitivity to both AMB and CAS, though significant differences in FLC susceptibility were not observed in deletion strains compared to the parental strains.

### Mannans are significantly enriched in the cell wall of a *hog1Δ* mutant.

The cellular flocculation and differential susceptibility of *ssk1Δ* and *hog1Δ* mutants to cell wall-perturbing agents indicate changes in the cell wall architecture in these strains. Therefore, we quantified the major carbohydrate components of the fungal cell wall such as glucan, mannan, and chitin. Our results revealed that mannans were significantly elevated in the *hog1Δ* mutant in the AR384 background (Fig. 4B). Additionally, the AR384 strain contained a higher content of mannans than the AR389 and 1184/P/15 strains. It is important to point out that the AR384 strain belongs to the African clade while AR389 and 1184/P/15 strains belong to the South Asian clade. The biological implications of elevated mannans in the *hog1Δ* mutant in the AR384 background are not well understood. It may be a clade-specific function of Hog1. Therefore, more experimentation is needed to further investigate the exact mechanism of action of Hog1 in the AR384 strain of C. auris. There was no significant difference in glucan or chitin contents between all C. auris strains.

### The C. auris Ssk1 response regulator mediates phosphorylation of Hog1 MAP kinase.

**FIG 5:**
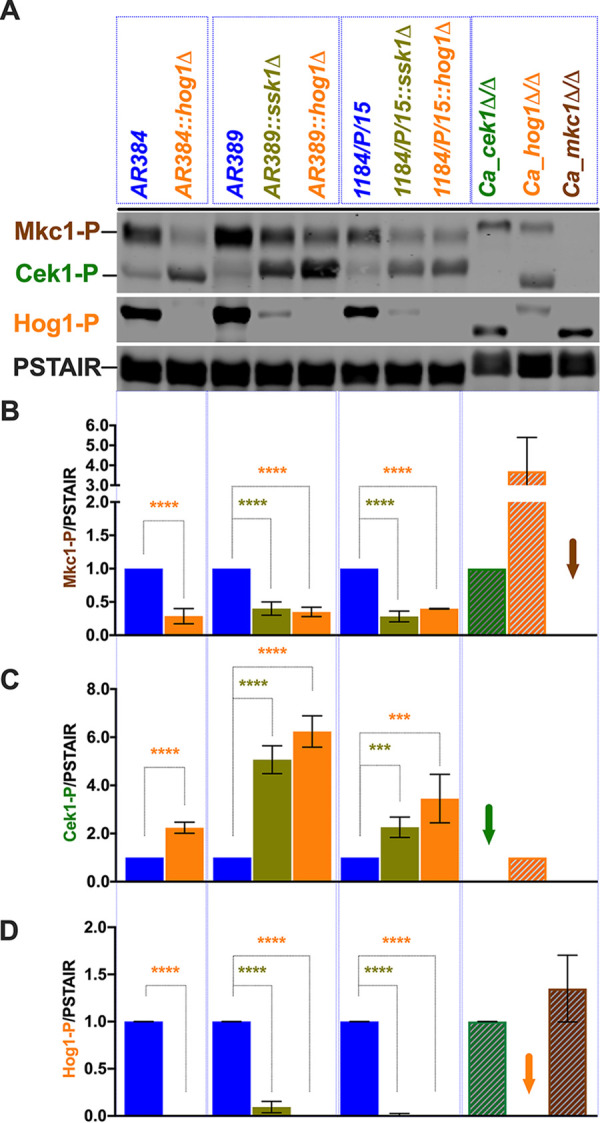
Ssk1-mediated Hog1 pathway activation positively regulates Mkc1 cell integrity pathway. (A) Logarithmically growing Candida auris cultures were washed once with ice-cold water, and whole-cell extracts for immunoblotting were prepared by the trichloroacetic acid (TCA) method. Extracts corresponding to an OD_600_ of 1.0 were fractionated by 12% SDS-PAGE and subjected to immunoblotting as indicated, using commercially available antibodies for the activated phosphorylated MAP kinases Mkc1-P and Cek1-P (phospho-p44/42 MAPK [Erk1/2] [Cell Signaling Tech]) and Hog1-P (phospho-p38; Cell Signaling Tech). Reprobing the blots with the PSTAIR antibodies (Sigma) recognizing Cdc28 served as a loading control. Protein bands were visualized using an Odyssey CLx scanner (Li-Cor). (B to D) Densitometry was performed using Image Studio software (Li-Cor). Data are expressed as fold change normalized to the PSTAIR (Cdc28) loading control; extracts from parent strains were set to 1. Results are from three to five independent biological samples (±SD; ***, *P* ≤ 0.0005; ****, *P* < 0.0001). Proteins from C. albicans *cek1*Δ/Δ, *hog1*Δ/Δ, and *mkc1*Δ/Δ strains were used as controls, and the loss of respective signal is shown with the arrow.

**FIG 6:**
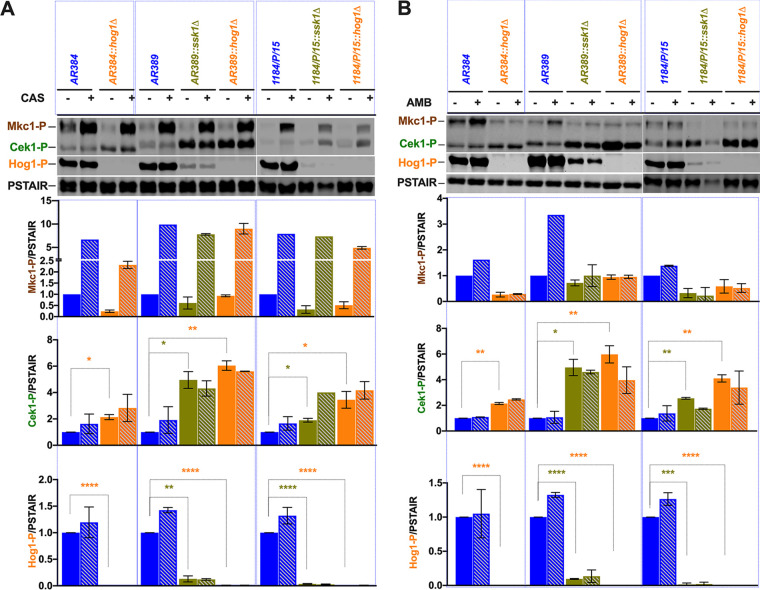
Antifungal drugs differentially modulate MAPK responses in C. auris. (A) Caspofungin treatment modulates MAPK signaling pathways in C. auris. Logarithmically growing Candida auris cultures were treated with 100 ng/ml caspofungin for 15 min and washed once with ice-cold water, and whole-cell extracts were prepared for immunoblotting as described in the legend to Fig. 5A. Densitometry was performed using Image Studio software (Li-Cor). Data are expressed as fold change normalized to the PSTAIR (Cdc28) loading control; extracts from untreated control strains were set to 1. Results are from two independent biological samples (±SD; *, *P* ≤ 0.05; **, *P* ≤ 0.005; ***, *P* ≤ 0.0005; ****, *P* < 0.0001). Striped columns indicate the drug (CAS) treatment. (B) Amphotericin B treatment modulates MAPK signaling pathways in C. auris. Logarithmically growing Candida auris cultures were treated with 500 ng/ml amphotericin B for 15 min and washed once with ice-cold water, and whole-cell extracts for immunoblotting were prepared as described above and subjected to immunoblotting as described for panel A. Striped columns indicate drug (AMB) treatment conditions.

Hog1 is a stress-activated MAP signaling kinase that controls the osmostress response (38, 39). The Ssk1 response regulator is part of TCS acting upstream of Hog1. Of note, Ssk1 is critical for Hog1 activation under oxidative stress (16, 40, 41). To determine the role of Ssk1 inactivation in MAPK signaling pathways, we performed Western blotting of cell extracts from parental strains and deletion mutants in the presence or absence of antifungal drugs (Fig. 5 and 6). Ssk1 was essential for activating Hog1 upon CAS and AMB treatment, since activated Hog1-P was reduced by about 3-fold (Fig. 5 and 6). Although we noticed some variations in phosphorylation signals in different strain backgrounds and in the presence or absence of CAS and AMB (Fig. 5 and 6), the data demonstrate that C. auris Ssk1 acts upstream of the Hog1 kinase module and transduces signals through Hog1 activation, as shown for other fungal pathogens (15).

### Amphotericin B and caspofungin differentially regulate MAPK signaling in C. auris.

Mkc1, Hog1, and Cek1 are three distinct key MAPK modules guarding cell wall integrity, surface remodeling, and stress adaptation as well as morphogenesis and filamentation (42, 43). Further, these MAPK pathways have been implicated in virulence of several pathogenic fungi (44–48). To check the activation status of MAPK pathways in C. auris strains, we immunodetected activated Hog1-P and Mkc1-P in cell extracts from strains (Fig. 6). We used anti-Cdc28 PSTAIR antibodies to confirm equal loading. Surprisingly, 2- to 3-fold-lower levels of activated Mkc1-P were detected in all strain backgrounds upon loss of Ssk1, compared to the parental controls (Fig. 6). As expected, no Hog1-P signal was detected in *hog1*Δ cells in any of the strain backgrounds. The *ssk1*Δ deletion also strongly impaired Hog1-P activation (Fig. 6). Because we noticed a difference in migration of Hog1 (49), Mkc1, and Cek1 between C. auris and C. albicans (Fig. 6), we employed extracts from C. albicans *cek1*Δ/Δ, *hog1*Δ/Δ, and *mkc1*Δ/Δ strains as controls (50) to verify antibody specificity.

To gain more insight about CAS and AMB effects on the MAPK activation in C. auris, we performed immunoblotting of protein extracts from logarithmically growing cells with or without a 30-min treatment with AMB (500 ng/ml) or CAS (100 ng/ml). We immunoblotted with phospho-p44/42 and phospho-p38 antibodies, detecting the activated isoforms Mkc1-P, Cek1-P, and Hog1-P, respectively. After reprobing with PSTAIR antibodies without prior stripping, we used IRDye-conjugated secondary antibodies for detection. The data showed increased levels of activated Mkc1-P appearing in all C. auris strains upon CAS treatment (Fig. 6). In contrast, only the parental strains AR384, AR389, and 1184/P/15 responded to AMB by increasing Mkc1-P levels. In contrast, *ssk1*Δ and *hog1*Δ cells failed to induce Mkc1 activation following AMB treatment (Fig. 6). Therefore, activated Mkc1-P appears critical for cell integrity in response to CAS and AMB in C. auris. Surprisingly though, no changes in Hog1-P levels were observed in either AMB- or CAS-treated cells, perhaps because a high basal level of Hog1-P activation was already present. We also determined the activation of Cek1 in response to both CAS and AMB. Indeed, Cek1-P increased 2- to 4-fold in all strains, except for the AR389 *ssk1*Δ and *hog1*Δ mutants responding to CAS. Interestingly, we noticed marked differences of Cek1-P in SDS-PAGE mobility between the C. auris strain belonging to the African clade (AR384) and those belonging to the South Asian clades (AR389 and 1184/P/15). Of note, our results indicate a strongly increased constitutive activation of Cek1 in *ssk1*Δ and *hog1*Δ mutants in South Asian clade strains (Fig. 6). Taken together, these data suggest a potential and extensive cross talk between the Mkc1, Hog1, and Cek1 MAPK signaling pathways in C. auris facing antifungal drug stress. Our data show that C. auris Ssk1 operates upstream of both Hog1 and Mkc1 and is important for transducing the environmental signals to both Hog1 and Mkc1. Furthermore, Ssk1 shows a direct or indirect genetic link to the Cek1 pathway, because *ssk1*Δ mutants display enhanced constitutive levels of Cek1-P.

**FIG 7:**
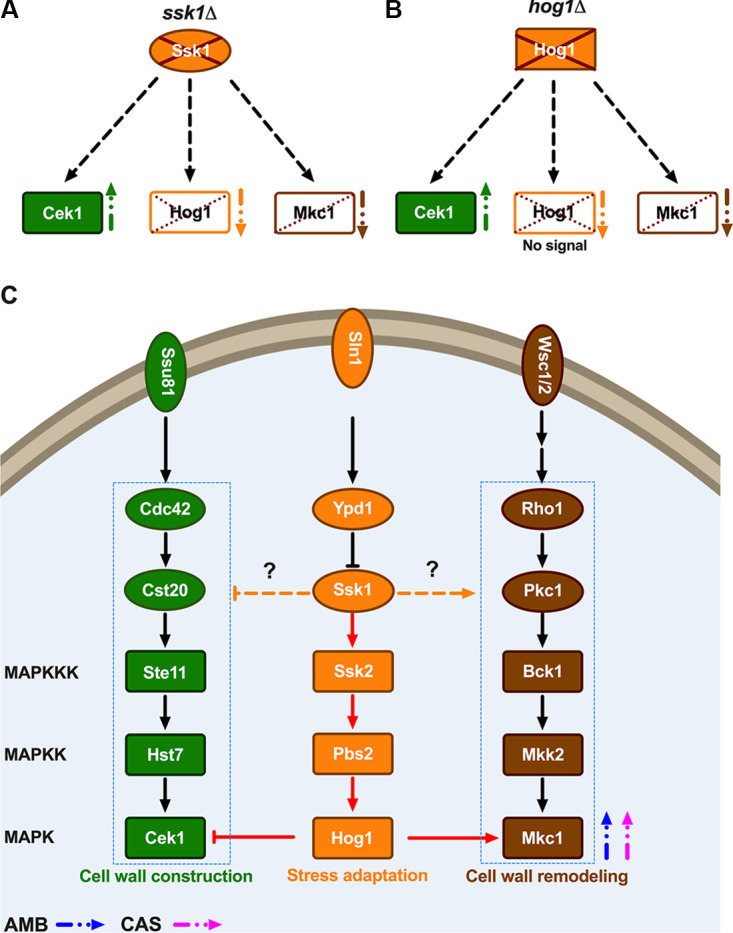
Model for MAPK signaling cross talk in response to antifungal drugs in C. auris. Linear MAP kinase pathways (MAPKKK→MAPKK→MAPK) are represented in rectangles; upstream components are in ovals. Cek1 (green)-, Hog1 (orange)-, and Mkc1 (brown)-containing MAPK pathways are color coded. Open rectangles indicate either absence or reduced phosphorylation. (A) Lack of Ssk1 protein increases p-Cek1 (upward green arrow), reduction or absence of p-Hog1 (downward orange arrow), and partial reduction of p-Mkc1 (downward brown arrow). (B) Absence of *HOG1* increases p-Cek1 (upward green arrow) but reduces p-Mkc1 (downward brown arrow). (C) MAPK signaling pathways such as Cek1, Hog1, and Mkc1 are illustrated. Modulation of signaling is indicated with either stimulatory arrows (→) or inhibitory arrows (⊣). Amphotericin B (AMB) and caspofungin (CAS) treatment triggers Mkc1 phosphorylation (blue and pink arrows, respectively). The Ssk1-driven pathway including Hog1 cross talk with Cek1 and Mkc1 is shown in red arrows. Activating or inhibitory roles of Ssk1 may involve direct or indirect interactions with Ste11 or Cek1 (p-Cek1) or with Bck1 (p-Mkc1) (orange dashed arrows with question marks).

Based on these observations, we propose a model (Fig. 7) describing a tightly wired, dynamic MAPK signaling network in C. auris. Although most MAPK components are conserved in C. auris, the data suggest that MAPK signaling in C. auris is more complex. The pathways seem to engage in cross talk upon activation of a single linear pathway by cell integrity challenges such as CAS or by membrane lipid perturbations as triggered by AMB. Importantly, our data suggest a great degree of plasticity in MAPK responses in distinct clinical isolates, which may arise from strain-specific adaptations to individual host environments. Taken together, our data nonetheless suggest that targeting MAPK signaling in C. auris may offer therapeutic promise, since loss of either Ssk1 or Hog1 renders multidrug-resistant clinical C. auris isolates susceptible to both CAS and AMB.

## DISCUSSION

Whole-genome sequencing and epidemiological studies have classified C. auris isolates into four phylogenetically distinct clades (6), with a potential fifth clade emerging from Iran (7). The genetic diversity within each clade seems quite low, whereas, the interclade heterogeneity among C. auris isolates is rather extensive, with tens of thousands of distinct single nucleotide polymorphisms (SNPs) in the genomes (7). Interestingly many putative SNPs in *ERG11* and *FKS1* genes are associated with clinical azole and echinocandin resistance (8, 51).

Here, we present data about complex relationships between the C. auris genotype and its phenotype in MAPK kinase-dependent stress adaptation that also affects antifungal MDR phenotypes. The C. auris clinical isolates used in this study display variable phenotypic characteristics when cultivated under different growth conditions, including high temperature, and in the presence of oxidative stress or antifungal drugs. In the current study, we sought to understand the role of Ssk1 response regulator and the downstream Hog1 signaling kinase in stress and antifungal drug resistance. Of note, the role of C. auris Hog1 was recently reported to play a role in drug sensitivity, indicating that *hog1*Δ mutants are more CAS resistant. These observations were noted with three independent mutant isolates. Additionally, the *hog1Δ* mutant was found to be attenuated for virulence in a Caenorhabditis elegans model of infection (49). In contrast, our data show that both *ssk1*Δ and *hog1*Δ mutants display strongly increased susceptibility to both AMB and CAS as well as several known cell wall-perturbing agents. These differences could be attributable to distinct clade backgrounds of the C. auris strains used in each study. The antifungal susceptibility data presented here in at least two different clade backgrounds imply that Ssk1 and Hog1 play synergistic or at least additive functional roles in C. auris, because both AMB and CAS have distinct mechanisms of action (52). Another recent report has implied that the C. albicans Hog1 mediates resistance to AMB (53), but a possible link of the Ssk1 response regulator with Hog1 MAP kinase in mediating CAS resistance has not been explored. Interestingly, AMB binds to and sequesters membrane ergosterol (54). Hence, AMB is likely to cause massive sterol clustering or redistribution in the plasma membrane, thereby affecting membrane permeability and integrity (54). This would explain the observed AMB-driven activation of both Mkc1 and Hog1, as lipid changes also affect osmosensitivity. The fungal TCS and the Hog1 MAP kinase pathway are known to play important roles in the regulation of cell wall biosynthesis in C. albicans, S. cerevisiae, Cryptococcus neoformans, and Aspergillus fumigatus (44). Additionally, the fungal cell wall architecture and lipid membrane are the key target of antifungal drugs such as CAS and AMB, respectively (55). AMB most likely causes fungicidal ergosterol shifts and/or lipid redistribution in the plasma membrane, including a malfunctioning electrochemical gradient. It will therefore be very interesting to test in future experiments whether the alternative YPK1-YKR cell integrity signaling pathway controls AMB responses in C. auris (56, 57). Interestingly enough, YPK1 is activated by changes in the membrane lipid architecture and signals via Orm1 into the canonical Mkc1 cell integrity pathway (58).

CAS is a well-described inhibitor of fungal glucan synthesis. The success of CAS in the clinical setting is based on its fungicidal action, which comes with much lower toxicity than AMB. CAS blocks Fks1-mediated glucan deposition into the growing cell wall. Thus, it is therefore not surprising that CAS-resistant clinical C. auris isolates carry mutations and/or SNPs in *FKS1* mutational hot spots associated with CAS resistance (51, 59). Since CAS causes massive damage to the cell wall architecture, it is also not surprising that Mkc1 cell integrity is rapidly activated to enable compensatory responses.

Importantly, fungal adhesion may be a major virulence determinant for C. auris, owing to its propensity to adhere to human skin tissues (60). Of note, adhesion genes are also tightly controlled by MAPK as well as protein kinase A signaling in other fungal pathogens (61, 62). Indeed, preliminary transcriptome sequencing (RNA-seq) data for several C. auris clades demonstrate that adhesion is among the most highly regulated processes in many C. auris strains (data not shown). It will be exciting to dissect the molecular players and regulators driving adhesion of C. auris to biotic and abiotic surfaces. Nonetheless, based on the complex MAPK cross talk observed here, we believe that this may be beneficial for C. auris to adapt to host defense and allow for immune evasion. Although virulence and immune recognition are quite similar in C. auris compared to C. albicans (63), it is tempting to speculate that synergistic and dynamic cross-talks of MAPK networks may be implicated in promoting the appearance of panresistant isolates.

Our data suggest that Ssk1 and Hog1 appear to have distinct functions in different clinical isolates. These data suggest that the function of *SSK1* and *HOG1* varies with each strain, most likely owing to distinct or partial rewiring of upstream signaling components and/or the connection with downstream transcriptional regulators. Based on these observations, we hypothesize that this phenomenon of complex genetic interactions has been an evolutionary driver of variable pathway function in different strain backgrounds. We believe that this is more likely the rule rather than an exception, especially for commensal microbial pathogens that are under permanent selection pressure or immune surveillance. Although some master regulators of fundamental processes like filamentation may have been functionally conserved in both pathogenic and nonpathogenic fungi (64), a constant selection pressure or host immune surveillance may drive evolutionary adaptation. This must be the case for pathogens like C. auris, whose emergence and appearance in humans may have involved distinct animal species and environmental habitats (65). Indeed, a recent study strongly supports this notion (66). Remarkably, loss of several master regulators of morphogenesis and biofilm formation, including *BCR1*, *UME6*, *EFG1*, and *BRG1*, shows highly divergent phenotypes even between the standard C. albicans laboratory strain SC5314 and several unrelated C. albicans clinical isolates, suggesting extensive rewiring of signaling networks under immune surveillance. Importantly, these observations strongly suggest that testing or quantifying drug resistance or virulence phenotypes of fungal deletion mutants should always include a series of fungal strains from different genetic backgrounds or clades within the same genus (66). In fact, this also applies to host immune recognition of fungal pathogens, as fungal strain variabilities accounted for dramatic differences concerning recognition of fungal pathogen-associated molecular patterns (PAMPs) by immune receptors like dectin-1 (67–69).

The Ssk1 response regulator transduces environmental stress signals to activate the Hog1 pathway in C. albicans and other fungal pathogens (16). Consistent with previous reports, our results show that Ssk1 is required for the phosphorylation of C. auris Hog1. Furthermore, our results demonstrate an active cross talk between Hog1 and the MAPK Mkc1 or Cek1 pathways. Of note, there are clade-specific differences in the mobility of Cek1 in C. auris in the African and South Asian clades. Cek1 from the South Asian clade has an insertion of a 10-residue stretch at the N terminus, which is found only in the African clade but not in any other *Candida* species, but deducing any functional impact will require further experiments.

Importantly, this cross talk is enhanced in the presence of antifungal drugs such as CAS or AMB (Fig. 5 and 6). Therefore, these data suggest a high connectivity and dynamics of MAPK signaling in C. auris, perhaps forming a dynamic network that ensures efficient responses and swift adaptation to environmental stimuli or host immune defense. Such a rewiring of signal transduction pathways would confer hypersensitivity on C. auris to pressure, as it may also come at a cost of fitness, which is often seen when a stress response is activated (70, 71). However, here rewiring may offer significant advantages to clinical isolates of C. auris to cope with antifungal drugs and immune surveillance. To avoid such fitness costs over extended periods, these adaptive mechanisms have to be dynamic and reversible, which may explain some of the phenotypic plasticity of various clinical isolates. For example, the C. auris strain 1184/P/15 is unable to cope with oxidative stress and is unable to grow at elevated temperature.

Finally, the functions of C. auris *SSK1* and *HOG1* appear different not only from other fungal pathogens but also within C. auris clades, and even in strains from the same clade. We show that *SSK1* and *HOG1* play critical roles in antifungal MDR, and this function appears to engage the Hog1, Mkc1, and Cek1 MAP kinase signaling. Remarkably, all of these MAPK pathways are guarding proper cell wall functions as well as surface architecture. Indeed, the fungal cell wall is critical for adhesion to abiotic and biotic surfaces such as the human skin. In fact, skin tissues offer an easily accessible substrate for growth and adhesion by C. auris and pose the single most important threat for person-to-person transmission (60). Since Ssk1, Mkc1, and Hog1 are differentially regulated upon various stress conditions, it will be interesting to test possible roles in the adhesion and colonization of human skin tissues. Based on the data presented here, we propose that Ssk1 may represent a reasonable antifungal target for several reasons. First, deletion of *SSK1* restores the antifungal susceptibility to AMB and CAS of MDR C. auris strains resistant to AMB and CAS. Second, the advantage of targeting nonessential genes holds a reduced risk of rapid emergence of drug-resistant mutants. Finally, Ssk1 is not conserved in humans, suggesting that adverse drug toxicity due to inhibition of human targets is less likely, though it would not go beyond adverse effects intrinsically found in any drug discovery process. Thus, we propose that the C. auris fungal two-component system, a signal transduction pathway conserved in most fungi, holds promise for developing new antifungals, since it controls key pathogenic traits such as virulence and anti-infective drug susceptibilities.

## MATERIALS AND METHODS

### Candida auris strains and growth conditions.

Candida auris clinical isolates and mutant strains were grown in rich medium (YPD; 1% yeast extract, 2% peptone, and 2% dextrose) at 30°C with shaking at 200 rpm. Logarithmic-phase cells were obtained by growing overnight cultures in fresh YPD medium for 4 h at 30°C. Nourseothricin at 200 μg/ml was used as a selection marker for C. auris. Two percent agar was added to the plates. The C. auris strains and primers used in this study are listed in Tables S1 and S2 in the supplemental material.

10.1128/mSphere.00973-20.7 TABLE S2 List of plasmids used in this study. Download Table S2, DOCX file, 0.02 MB. Copyright © 2020 Shivarathri et al. 2020 Shivarathri et al. This content is distributed under the terms of the Creative Commons Attribution 4.0 International license.

### Fungal deletion mutant construction.

The deletion of both *SSK1* (B9J08_005450) and *HOG1* (B9J08_004369) was performed by using the modified fusion PCR method (29). Briefly, upstream and downstream flanking regions of the *SSK1* and *HOG1* genes were amplified using appropriate primers (Table S2), adding 20-bp constant overlap sequences U1 and D1 at the 3′ end of upstream and 5′ end of downstream regions, respectively. The dominant marker *NAT1* was amplified from the plasmid pSFS3b (72) using the primers NAT1_fwd_U1 and NAT1_rev_D1 covering constant complementary 20-bp sequences U1 and D1. PCR-amplified upstream, downstream, and *NAT1* marker fragments were gel purified and subjected to fusion PCR using *Ex Taq* polymerase (TaKaRa) (Fig. S1). The conditions and settings used for the fusion PCR were as follows: for 50-μl reaction volume, 1× *Ex Taq* buffer, 0.2 μM deoxynucleoside triphosphates (dNTPs), 0.5 μM (each) primer, 3 μl marker fragment, 1.25 μl each flanking homology fragment, and 0.25 μl *Ex Taq* polymerase; 98°C for 5 min; 30 cycles of 98°C for 20 s, 50°C for 30 s, and 72°C for 1 min (for 1-kb fragment); and a final extension at 72°C for 10 min. The purified gene deletion constructs (upstream-NAT1-downstream) were used to transform into C. auris clinical isolates (Fig. S1). Transformation of C. auris was done via the lithium acetate (LiAc)/single-stranded (SS) carrier DNA/polyethylene glycol (PEG) method as described previously (73). Strains were initially verified by colony PCR and then later genomic DNA PCR to confirm correct genomic integration of the deletion cassette (5C/3C primers), as well as the loss of the coding sequence (internal primers).

### Growth and phenotypic profiling.

To study the effect of *in vitro* stressors and antifungal drugs, the C. auris strains were grown in YPD broth overnight at 30°C. From an overnight culture, cells corresponding to an optical density at 600 nm (OD_600_) of 0.1 were inoculated into fresh YPD broth with or without caspofungin (100 ng/ml), amphotericin B (500 ng/ml), calcofluor white (50 μg/ml), caffeine (50 mM), and SDS (0.05%). Absorbance was recorded in an H1 synergy plate reader at regular intervals for a period of 24 h, and the OD_600_ values were plotted versus time. Additionally, the phenotypic characterization of C. auris mutants was done via serial dilution spotting assays on YPD agar plates. Equal volumes (3 μl) of 10-fold serial dilutions of logarithmically growing C. auris strains were spotted onto YPD agar plates containing different stress agents such as temperature stress (42°C), hydrogen peroxide (H_2_O_2_, 7.5 mM), caffeine (50 mM), SDS (0.05%), calcofluor white (CFW, 50 μg/ml), caspofungin (CAS, 100 and 200 ng/ml), amphotericin B (AMB, 0.5 and 2 μg/ml), and fluconazole (FLC, 16 and 128 μg/ml). Colony growth was scored after 48 h and compared to the YPD agar control plate.

### Western blot analysis.

Logarithmically growing C. auris clinical isolates and mutants were treated with or without AMB (0.5 μg/ml) and CAS (100 μg/ml) for 10 min. After that, cultures were washed once with ice-cold water and whole-cell extracts were prepared by the trichloroacetic acid (TCA) method as described previously (50). Extracts corresponding to 0.5 OD_600_ were fractionated by 12% SDS-PAGE and blotted for proteins as indicated. Signals from the same whole-cell extracts were detected using antibodies for active phosphorylated MAP kinases. The commercial antibodies recognized phosphorylated Mkc1-P and Cek1-P (phospho-p44/42 MAPK [Erk1/2]; Cell Signaling Tech) and Hog1-P (phospho-p38; Cell Signaling Tech). Reprobing with PSTAIR antibody (Sigma) recognizing Cdc28 (B9J08_002497) served as a loading control. Protein bands on the nitrocellulose membrane were visualized using an Odyssey CLX scanner (Li-Cor). Quantification of the protein band intensity was performed by using Image Studio software (Li-Cor). The intensity ratios of phosphorylated versus loading control were used to generate heat maps in GraphPad Prism software.

### Fluorescein diacetate (FDA) uptake assay.

The kinetics of FDA uptake was carried out with a slightly modified protocol (50). Briefly, logarithmically growing C. auris strains were harvested at about 0.5 OD. Cells were resuspended and washed twice in 1 ml of FDA buffer (50 mM HEPES, pH 7.0, and 0.5 mM 2-deoxy-d-glucose) before supplementing with 50 nM FDA. A 200-μl volume of cell mixture with or without FDA was added to an optical-bottom 96-well black plate. The kinetics of FDA uptake was recorded every 5 min for 30 reads or until saturation was reached with simultaneous shaking of samples on the H1 Synergy plate reader with excitation and emission wavelengths of 485 and 535 nm, respectively. Data represent the mean fluorescence intensity over time. The slope was calculated using GraphPad Prism software.

### Flocculation assay.

Flocculation was determined by growing the C. auris strains to the late exponential growth phase in YPD broth at 30°C as previously reported for C. albicans (74). Equal-OD_600_ cells were transferred to separate culture tubes. The culture tubes were vigorously vortexed and allowed to settle for 5 to 10 min. Images were recorded after 5 to 10 min.

### Microscopy.

To visualize any changes in cellular morphologies due to the deletion of *SSK1* and *HOG1*, the C. auris strains were grown in YPD broth at 30°C. One milliliter of logarithmically growing cells was washed twice with phosphate-buffered saline (PBS) and fixed in 4% *p*-formaldehyde for 2 h. Fixed cells were washed, and images were taken with a Zeiss Axiovert 200 microscope at ×63 magnification. Approximately 100 to 200 cells were counted, and elongated and pseudohypha-like cells were represented as a percentage (scale bar = 5 μm).

### Quantification of cell wall components by flow cytometry.

Quantification of cell wall components by flow cytometry was performed as described previously (75). Briefly, logarithmically growing C. auris strains were washed and stained with concanavalin A-conjugated Texas Red, dectin-1/Fc+488, and calcofluor white to decorate mannans, glucan, and chitin, respectively. These triple-stained cells were measured in a BD Fortessa flow cytometer (BD Biosciences) to quantify the amount of chitin, glucan, and mannan using the BV421 (violet 405 nm, 50-mW power), fluorescein isothiocyanate (FITC) (blue 488-nm wavelength, 50-mW power), and Texas Red (red 640-nm wavelength, 40-mW power) lasers, respectively. A minimum of 10,000 events were recorded for each sample, and the data were analyzed using FlowJo software (FlowJo LLC). Unstained and single-stained samples served as controls, and the data were expressed as the mean fluorescence intensity from three independent experiments.

10.1128/mSphere.00973-20.3 FIG S3 Differential MAPK signaling response in different clades of C. auris. Logarithmically growing Candida auris cultures were washed once with ice-cold water, and whole-cell extracts for immunoblotting were prepared by the trichloroacetic acid (TCA) method. Extracts corresponding to 1 OD_600_ were fractionated by 12% SDS-PAGE and subjected to immunoblotting as indicated, using commercially available antibodies for the activated phosphorylated MAP kinases Mkc1-P and Cek1-P (phospho-p44/42 MAPK [Erk1/2] [Cell Signaling Tech]) and Hog1-P (phospho-p38; Cell Signaling Tech). Reprobing the blots with the PSTAIR antibodies (Sigma) recognizing Cdc28 served as a loading control. Protein bands were visualized using an Odyssey CLx scanner (Li-Cor). (B to D) Densitometry was performed using Image Studio software (Li-Cor). Data are expressed as fold change normalized to the PSTAIR (Cdc28) loading control; extract from the clade I (South Asia) strain was set to 1. Results are from three independent biological samples (±SD; *, *P* < 0.05; **, *P* < 0.01; ****, *P* < 0.0001). Proteins from C. albicans *cek1*Δ/Δ, *hog1*Δ/Δ, and *mkc1*Δ/Δ strains were used as controls, and the loss of respective signal is shown with the arrow. (E) Sequence alignment of C. auris Cek1 from clade I (South Asian strain B8441), clade II (East Asian strain B11220), clade III (South African strain B11221), and clade IV (South American strain B11243) using Clustal Omega (https://www.ebi.ac.uk/Tools/msa/clustalo/). Asterisks indicate conserved residues. The insertion of 10 amino acids (QNQNQSQSQH) was found in clade I strains (blue letters) and missing in clade III and IV strains. Download FIG S3, TIF file, 1.1 MB. Copyright © 2020 Shivarathri et al. 2020 Shivarathri et al. This content is distributed under the terms of the Creative Commons Attribution 4.0 International license.

10.1128/mSphere.00973-20.4 FIG S4 Original digital images of immunoblots used to assemble Fig. 5. Odyssey settings were identical for all Western blots subjected to imaging. The yellow boxes indicate the sections cropped from the original digital images of individual immunoblots to compile Fig. 5. Sections in Fig. 5 obtained from independent immunoblots are separated by white spaces. Phospho-p44/42 MAPK (Erk1/2; Cell Signaling Tech) antibody recognizes both phosphorylated Mkc1-P and Cek1-P at approximately 70 kDa and 52 kDa, respectively. The phospho-p38 antibody (Cell Signaling Tech) recognizes phosphorylated Hog1-P at approximately 49 kDa and 45 kDa in C. auris and C. albicans, respectively. The PSTAIR antibody (Sigma) detects Cdc28 at 36 kDa. Download FIG S4, TIF file, 0.3 MB. Copyright © 2020 Shivarathri et al. 2020 Shivarathri et al. This content is distributed under the terms of the Creative Commons Attribution 4.0 International license.

10.1128/mSphere.00973-20.5 FIG S5 Original digital images of immunoblots used to assemble Fig. 6. Odyssey settings were identical for all Western blots subjected to imaging. The yellow boxes indicate the sections cropped from the original digital images of individual immunoblots to compile Fig. 4 and 5. Sections in Fig. 4 and 5 obtained from independent immunoblots are separated by white spaces. Phospho-p44/42 MAPK (Erk1/2; Cell Signaling Tech) antibody recognizes both phosphorylated Mkc1-P and Cek1-P at approximately 70 kDa and 52 kDa, respectively. The phospho-p38 antibody (Cell Signaling Tech) recognizes phosphorylated Hog1-P at approximately 45 kDa. The PSTAIR antibody (Sigma) detects Cdc28 at 36 kDa. (A) The original uncropped immunoblots which were used to assemble Fig. 6A. (B) The original uncropped immunoblots which were used to assemble Fig. 6B. Download FIG S5, TIF file, 1.7 MB. Copyright © 2020 Shivarathri et al. 2020 Shivarathri et al. This content is distributed under the terms of the Creative Commons Attribution 4.0 International license.

10.1128/mSphere.00973-20.8 TABLE S3 List of primers used in this study. Download Table S3, DOCX file, 0.02 MB. Copyright © 2020 Shivarathri et al. 2020 Shivarathri et al. This content is distributed under the terms of the Creative Commons Attribution 4.0 International license.
