# Supplementary figures and images for: The Two-Component Response Regulator Ssk1 and the Mitogen-Activated Protein Kinase Hog1 Control Antifungal Drug Resistance and Cell Wall Architecture of Candida auris
Source: mSphere. 2020 Oct 14;5(5):e00973-20. doi: 10.1128/mSphere.00973-20 (PMC7565899; doi:10.1128/mSphere.00973-20)

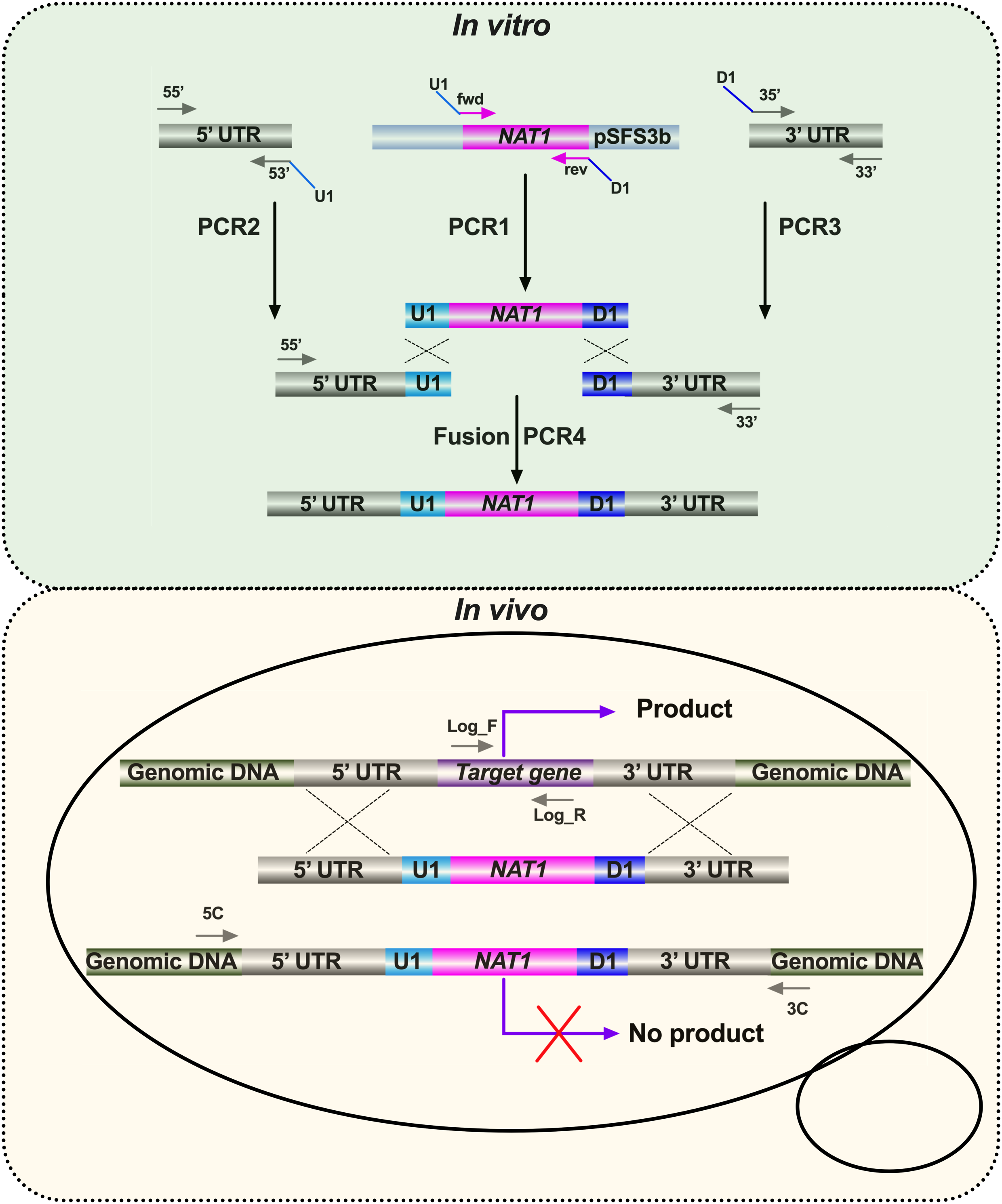

Supplement: FIG S1 [file mSphere.00973-20-sf001.tif]

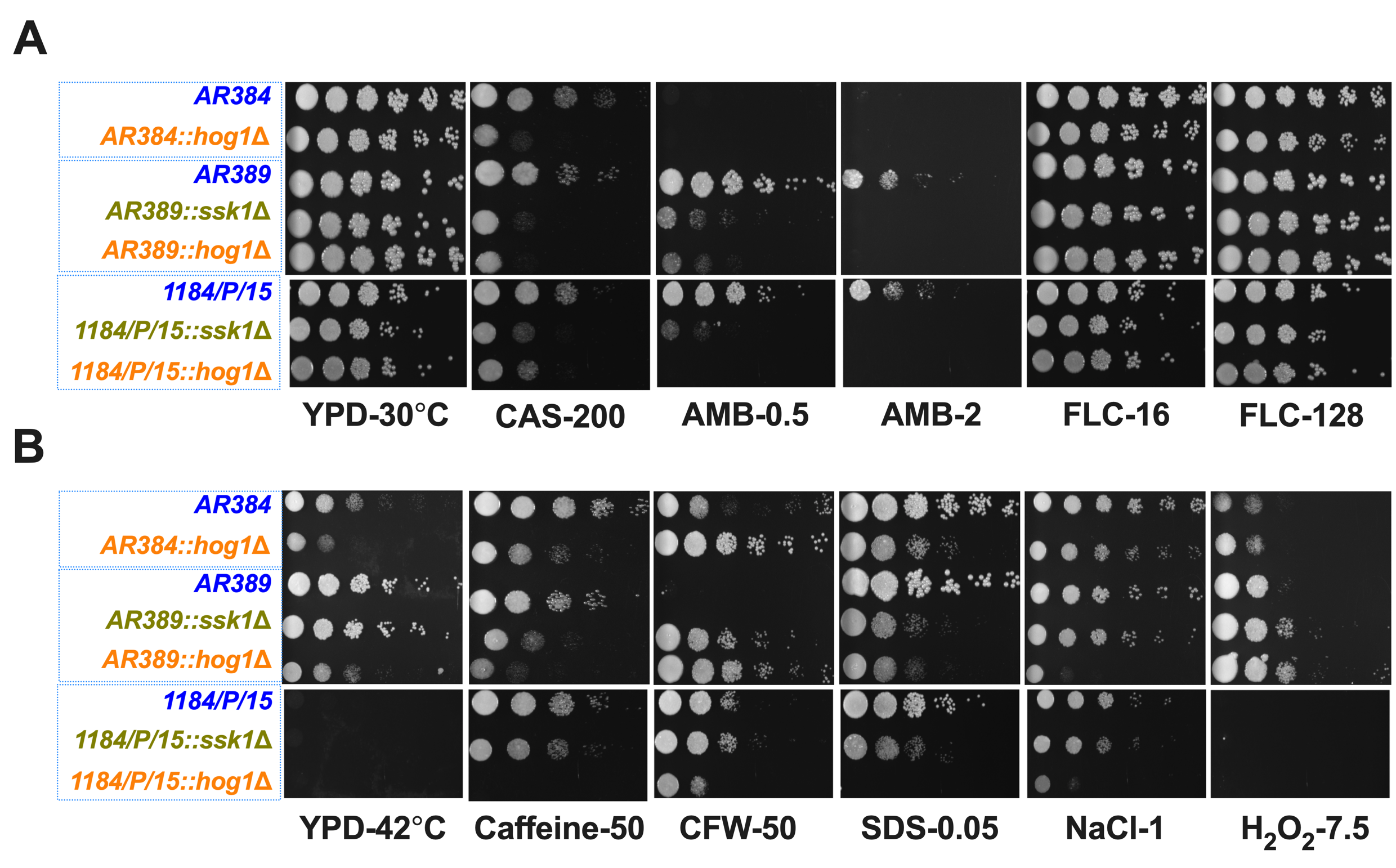

Supplement: FIG S2 [file mSphere.00973-20-sf002.tif]

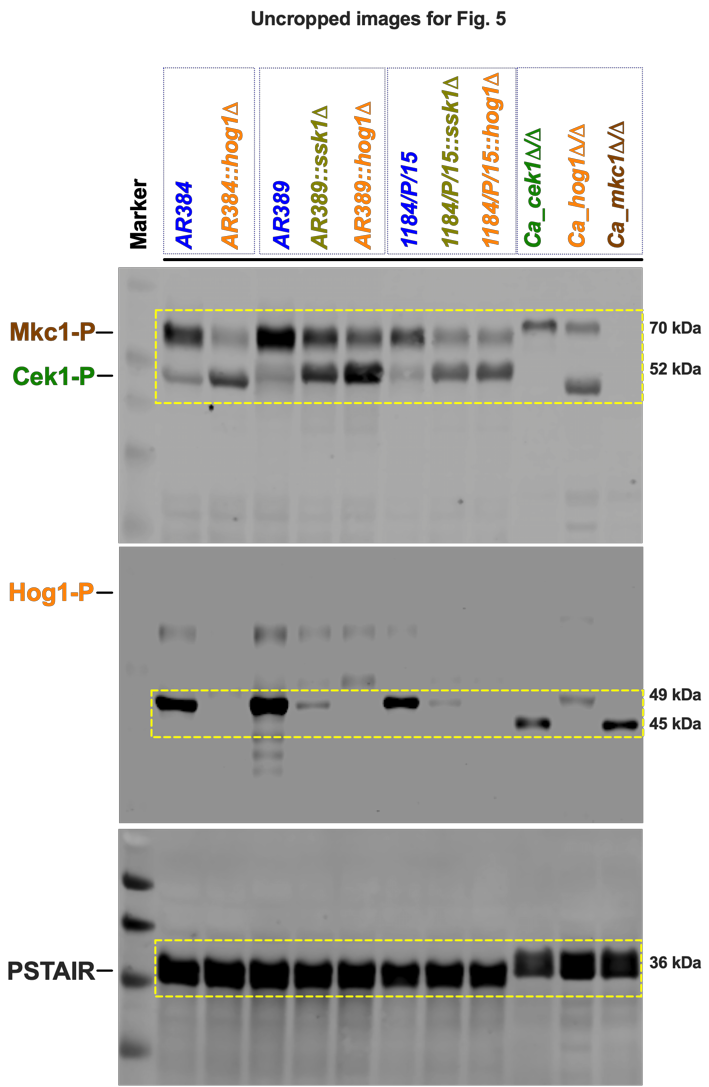

Supplement: FIG S4 [file mSphere.00973-20-sf004.tif]

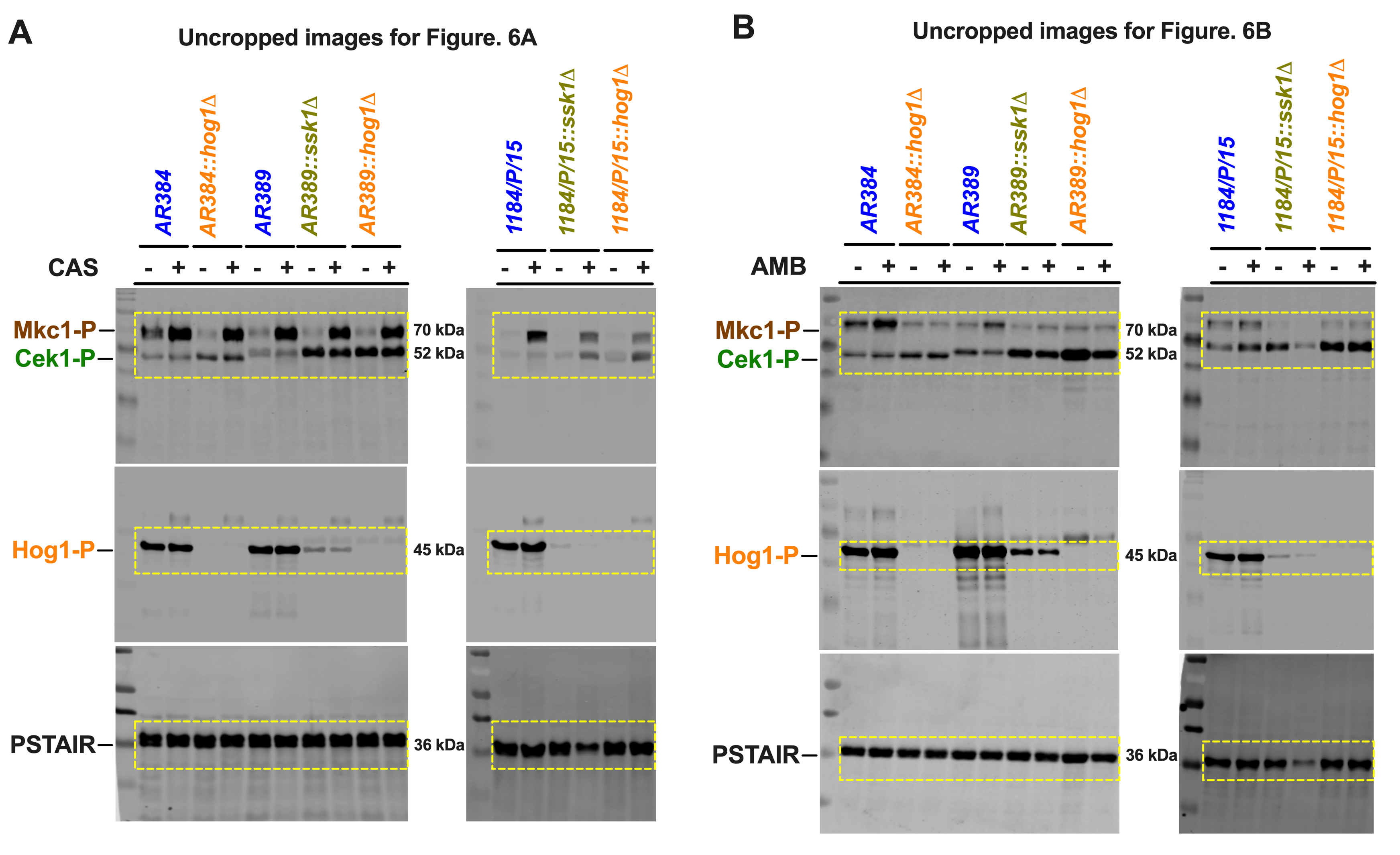

Supplement: FIG S5 [file mSphere.00973-20-sf005.tif]
