# Supplementary material for: The Two-Component Response Regulator Ssk1 and the Mitogen-Activated Protein Kinase Hog1 Control Antifungal Drug Resistance and Cell Wall Architecture of Candida auris
Source: mSphere. 2020 Oct 14;5(5):e00973-20. doi: 10.1128/mSphere.00973-20 (PMC7565899; doi:10.1128/mSphere.00973-20)
Supplement: TABLE S1 [file mSphere.00973-20-st001.docx]

**Table S1. *Candida auris and C. albicans* strains used in this study**

| **Name** | **Parent** | **Genotype** | **Clade** | **Collected from** | **Reference** | **Figures** |
| --- | --- | --- | --- | --- | --- | --- |
| ***C. auris*** | | | | | | |
| *ARBank0384* |  |  | III, Africa | CDC-USA |  | Figures 2, 3, 4, 5, 6 |
| *ARBank0385* |  |  | IV, South America | CDC-USA |  |  |
| *ARBank0389* |  |  | I, South Asia | CDC-USA |  | Figures 2, 3, 4, 5, 6 |
| *ARBank1097* |  |  | Iran | CDC-USA |  |  |
| *CBS10913* |  |  | II, East Asia | VPCI-India |  |  |
| *1184/P/15* |  |  | I, South Asia | VPCI-India | This study | Figures 2, 3, 4, 5, 6 |
| *AR384::hog1∆* | *AR0384* | *hog1∆::NAT1* | III, Africa | PHRI-USA | This study | Figures 2, 3, 4, 5, 6 |
| *AR389::ssk1∆* | *AR0389* | *ssk1∆::NAT1* | I, South Asia | PHRI-USA | This study | Figures 2, 3, 4, 5, 6 |
| *AR389::hog1∆* | *AR0389* | *hog1∆::NAT1* | I, South Asia | PHRI-USA | This study | Figures 2, 3, 4, 5, 6 |
| *1184/P/15::ssk1∆* | *1184/P/15* | *ssk1∆::NAT1* | I, South Asia | PHRI-USA | This study | Figures 2, 3, 4, 5, 6 |
| *1184/P/15::hog1∆* | *1184/P/15* | *hog1∆::NAT1* | I, South Asia | PHRI-USA | This study | Figures 2, 3, 4, 5, 6 |
| ***C. albicans*** | | | | | | |
| *cek1*∆*/*∆ | SC5314 | *cek1*∆*/cek1*∆::FRT |  |  | (1) |  |
| *hog1*∆*/*∆ | SC5314 | *hog1*∆*/hog1*∆::FRT |  |  | (1) |  |
| *mkc1*∆*/*∆ | SC5314 | *mkc1*∆*/mkc1*∆::FRT |  |  | (1) |  |

**Supplementary references**

1. Shivarathri R, Tscherner M, Zwolanek F, Singh NK, Chauhan N, Kuchler K. 2019. The Fungal Histone Acetyl Transferase Gcn5 Controls Virulence of the Human Pathogen Candida albicans through Multiple Pathways. Sci Rep 9:9445.

2. Reuss O, Vik A, Kolter R, Morschhauser J. 2004. The SAT1 flipper, an optimized tool for gene disruption in Candida albicans. Gene 341:119-27.

3. Tscherner M, Stappler E, Hnisz D, Kuchler K. 2012. The histone acetyltransferase Hat1 facilitates DNA damage repair and morphogenesis in Candida albicans. Mol Microbiol 86:1197-214.
