# Supplementary material for: The Two-Component Response Regulator Ssk1 and the Mitogen-Activated Protein Kinase Hog1 Control Antifungal Drug Resistance and Cell Wall Architecture of Candida auris
Source: mSphere. 2020 Oct 14;5(5):e00973-20. doi: 10.1128/mSphere.00973-20 (PMC7565899; doi:10.1128/mSphere.00973-20)
Supplement: TABLE S2 [file mSphere.00973-20-st002.docx]

**Table S2. Plasmids used in this study**

| **Plasmids** | **Parent** | **Relevant inserts and cloning sites** | **Reference** |
| --- | --- | --- | --- |
| pSFS2a |  |  | (2) |
| pSFS3b | pSFS2a | FRT-FLP-NAT1-FRT-BglII | (3) |
