# Supplementary material for: The Two-Component Response Regulator Ssk1 and the Mitogen-Activated Protein Kinase Hog1 Control Antifungal Drug Resistance and Cell Wall Architecture of Candida auris
Source: mSphere. 2020 Oct 14;5(5):e00973-20. doi: 10.1128/mSphere.00973-20 (PMC7565899; doi:10.1128/mSphere.00973-20)
Supplement: TABLE S3 [file mSphere.00973-20-st003.docx]

**Table S3. Primers used in this study**

Gene deletion oligonucleotides in 5’-3’ direction used in the *NAT1* fusion method

| **Name** | **Sequence (5'→3')** | **Reference** |
| --- | --- | --- |
| NAT1_UP_F | gcagggatgcggccgctgacAGCTTGCCTCGTCCCCGCCG | This study |
| NAT1_Dn_R | ccgctgctaggcgcgccgtgCTGGATGGCGGCGTTAGTATCG | This study |
| 55_CauSSK1 | ACTCTCTTTGGCCAATTGCG | This study |
| 53_CauSSK1 | gtcagcggccgcatccctgcGTGGATGATTGGTGAAGATGAT | This study |
| 35_CauSSK1 | cacggcgcgcctagcagcggCGGTCTGTGTTGTCTATACT | This study |
| 33_CauSSK1 | TGCGGTACTCTGATGAACTC | This study |
| Int1_CauSSK1_F | CTAGCTCGGCAGTTCGATCCT | This study |
| Int1_CauSSK1_R | GATAGGAGATAGACGGTGCG | This study |
| 5C_CauSSK1_F | GAAGATGACGACGAGGAGTTC | This study |
| 3C_CauSSK1_R | CTTCATTGGTTGCCAGGCGAT | This study |
| 55_CauHOG1 | ATAGATACGCTGCGAAATGC | This study |
| 53_CauHOG1 | gtcagcggccgcatccctgcTAACGAAGCAGTTAGTGGCT | This study |
| 35_CauHOG1 | cacggcgcgcctagcagcggGATACTTGAGTCAGATAGAC | This study |
| 33_CauHOG1 | GACGGTGGAGTTTGGTGTTG | This study |
| Int1_CauHOG1_F | CATGGGTGCGTTTGGACTTG | This study |
| Int1_CauHOG1_R | CAGACCACAAATCCACCTCAG | This study |
| 5C_CauHOG1_F | GCTAGAAGGTGAAGTTGATTC | This study |
| 3C_CauHOG1_R | GCATAGTTTGCAAGTGAATC | This study |
